# Pneumococcal Competence Coordination Relies on a Cell-Contact Sensing Mechanism

**DOI:** 10.1371/journal.pgen.1006113

**Published:** 2016-06-29

**Authors:** Marc Prudhomme, Mathieu Berge, Bernard Martin, Patrice Polard

**Affiliations:** Laboratoire de Microbiologie et Génétique Moléculaires, Centre de Biologie Integrative, Université de Toulouse, CNRS, UPS, France; Max Planck Institute for Terrestrial Microbiology, GERMANY

## Abstract

Bacteria have evolved various inducible genetic programs to face many types of stress that challenge their growth and survival. Competence is one such program. It enables genetic transformation, a major horizontal gene transfer process. Competence development in liquid cultures of *Streptococcus pneumoniae* is synchronized within the whole cell population. This collective behavior is known to depend on an exported signaling Competence Stimulating Peptide (CSP), whose action generates a positive feedback loop. However, it is unclear how this CSP-dependent population switch is coordinated. By monitoring spontaneous competence development in real time during growth of four distinct pneumococcal lineages, we have found that competence shift in the population relies on a self-activated cell fraction that arises *via* a growth time-dependent mechanism. We demonstrate that CSP remains bound to cells during this event, and conclude that the rate of competence development corresponds to the propagation of competence by contact between activated and quiescent cells. We validated this two-step cell-contact sensing mechanism by measuring competence development during co-cultivation of strains with altered capacity to produce or respond to CSP. Finally, we found that the membrane protein ComD retains the CSP, limiting its free diffusion in the medium. We propose that competence initiator cells originate stochastically in response to stress, to form a distinct subpopulation that then transmits the CSP by cell-cell contact.

## Author Summary

Development of competence for genetic transformation by cultures of pneumococcal cells has been considered till now as a classic example of quorum sensing, whereby a culture attaining a sufficient cell density detects a diffusible signaling molecule (in this case, Competence-Stimulating Peptide (CSP)) and switches en masse to a distinct physiological state. We find that the competence shift is dictated not by cell density but by growth for a time allowing emergence of a competence-initiator sub-population, and spreads by transmission of CSP through cell contact. This behaviour reflects the survival benefits of allowing subsets of the population to respond to environmental stress by generating signalling capacity, which prepares the entire population for a rapid and appropriate response to threatening conditions.

## Introduction

Under certain circumstances, single bacterial cells can sense environmental conditions and stimulate collective behavior by using exported signaling molecules that act as auto-inducers (AI). The two first processes found to be stimulated by AI sensing were luminescence in *Vibrio fischeri* [1] and competence for transformation in *Streptococcus pneumoniae* (the pneumococcus) [2]. Other examples of collective behavior have since been found [3–5]. These AI-based systems clearly differ in their mechanism. The first that has been defined is the Quorum Sensing (QS). It was proposed to take place by a rise in the concentration of a diffusible AI to a threshold level at which it induces the entire population to switch synchronously to a new gene expression program [6]. In particular, the QS mechanism implies that induction depends on the population achieving a given cell density (quorum) and on freely diffusing AI being produced at similar rates by all cells. The original QS model has been modified to take into account environmental parameters and the relative benefits for cells as individuals or as a group. The Diffusion Sensing mechanism includes the rate of loss of the AI in an open space [7], while the Efficiency Sensing mechanism takes account of cell distribution in complex environments [8]. Inclusion of these and other parameters has considerably increased the complexity of the original QS model [9]. Furthermore, the cost of each AI-based system and the specific purpose of the inducible genetic program are other important parameters that could have distinctly shaped their mechanism [10]. One such program, genetic competence in pneumococci, has long been thought to operate according to a QS model. This assumption has been challenged but without a clear alternative model emerging [11,12]. Here, we have studied in detail how the spontaneous development of competence for genetic transformation is coordinated throughout the population in planktonic pneumococcal culture.

Competence for genetic transformation is a distinct physiological state during which most of the proteins enabling cells to take-up and integrate exogenous DNA into the genome are produced. This process is widespread throughout the bacterial kingdom, wherein it acts as a central driver of evolution by promoting horizontal gene transfer [13]. Although transformation in all species proceeds through the same general mechanism, competence differs sharply among species at many levels. First, except for a small set of proteins dedicated to specific steps of the transformation mechanism, the genes that make up the competence regulon are variable. Second, the regulatory networks controlling the expression of competence genes differ considerably. Third, the time of competence induction and its duration with respect to culture growth cycle are clearly distinct among species, as are the factors that cause competence induction. These marked similarities and differences lead to the current notion that competence is tightly integrated into the life-style of each bacterial species [13]. A prominent difference so far observed only in pneumococcus and some closely related species is the synchronous development of competence of the whole cell population grown in liquid medium [14–16].

Two major properties are known to characterize Pneumococcal competence: import and integration of external DNA, called transformation, and killing of non-competent siblings or close relatives, called fratricide (for review see: [13,17]). In combination, these processes promote horizontal gene transfer and genome plasticity in pneumococci [18,19]. In support of this view, several studies have demonstrated how rapidly the pneumococcus can modify its genotype and undergo diversification [20–22]. These adaptations contribute to evasion of vaccines, antibiotics and host immune defenses [23–25].

**Fig 1:**
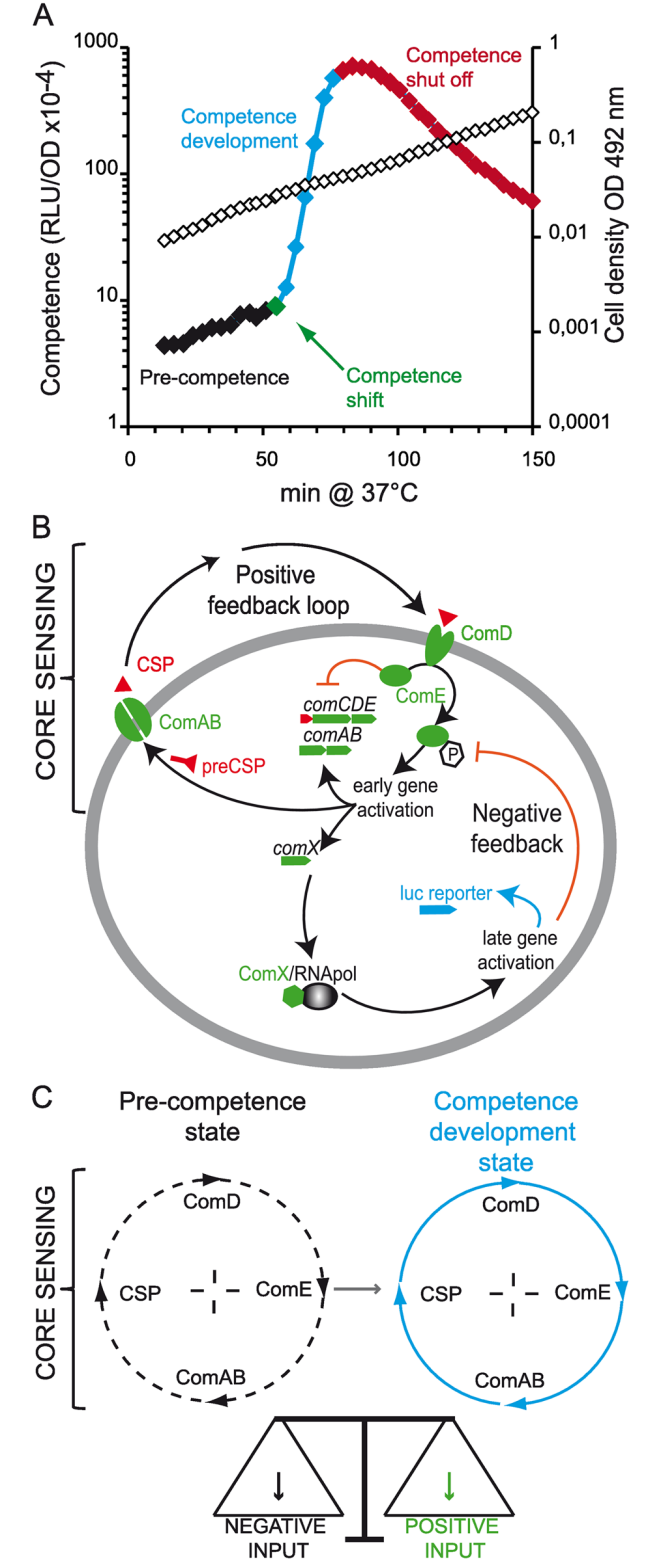
Spontaneous pneumococcal competence development. (A) The four steps of spontaneous competence development at the population level in the pneumococcus. A pre-culture stock of the R895 strain (which harbors the *luc* transcriptional fusion under the control of the *ssbB* late competence gene promoter) was inoculated at a 50 fold dilution as described in M&M. Open diamonds represent cell growth, closed diamonds the competence specific activity expressed in RLU.OD^−1^. Black diamonds correspond to the non competent state of the cell population (pre-competence), green diamonds correspond to the competence shift taken as the point of intersection between the non competence and the competence development phase, blue diamonds represent the period of competence development from which the rate can be calculated. Red diamonds correspond to competence shut off. RLU and OD reading were recorded in a Varioskan flash luminometer at 3-min intervals. (B) Schematic representation of pneumococcal competence regulation. *comC* encodes pre-CSP (depicted in red), which is exported as mature CSP (red triangles) by the *comAB* gene product. *comDE* encodes a two-component system made of ComD that senses CSP and, in turn, by phosphorylating the transcription regulator ComE. ComE~P activates a set of genes called early competence genes, pictured in green, with the exception of *comC* that is depicted in red. The products of the *com*AB and *com*CDE operons form a core sensor, named “ComABCDE”, which constitutes a positive feedback loop controlling competence induction (as depicted in C). The early genes also include *comX*, which encodes an alternative sigma factor (ComX) that directs the RNA polymerase core to a second set of competence genes, named the late competence genes. Amongst these, *dprA* shuts off competence, thus generating a negative feedback loop of competence. A transcriptional fusion between any early or late competence gene promotor with the luciferase gene (*luc*) will report on competence expression throughout the growth, as shown in A. (C) The ComABCDE core sensor of pneumococcal competence. In the pre-competence state, components of the ComABCDE core sensor are expressed at a basal level [16], insufficient to produce enough CSP for competence induction. This state is referred to as an idle mode shown by dotted arrows on the circular representation of the ComABCDE core sensor. Several external or internal inputs can negatively or positively impact the rate of synthesis and the stability of the components of the ComABCDE (for review see: [11,17]). When positive inputs dominate, the core sensor is activated and the CSP reaches a threshold value that shifts the cell into a competence development state. This activated mode of the core sensor is represented by full arrows.

In liquid cultures of Pneumococcus, competence develops transiently during exponential growth. Direct, continuous monitoring of competence gene expression [26] has revealed four consecutive steps in competence development, namely pre-competence, competence shift, competence development and shut-off (Fig 1A). The competence AI is a 17 amino-acid peptide called CSP (Competence Stimulating Peptide) [15]. It is at the heart of a positive feedback loop comprising 5 genes organized into two operons (Fig 1B). CSP is the product of the first gene of the *comCDE* operon, synthesized as a 41 amino acid precursor (pre-CSP) which is matured and exported by a dedicated membrane peptidase transporter, ComA/ComB, encoded by the *comAB* operon [17] (Fig 1B). Once CSP reaches a threshold concentration, it is sensed by the ComD membrane kinase of the two component system ComD/ComE. The membrane-bound ComD, activated by CSP interaction, mediates its autophosphorylation. In turn, ComD~P is assumed to transfer its phosphate to the ComE transcription regulator, switching it from a repressor to an activator which targets the *comAB* and *comCDE* operons and creates a positive feedback loop [27] (Fig 1B). Among the early competence genes activated by ComE~P is a pair of identical genes, *comX1* and *comX2*, which encode ComX, an alternative sigma-factor that activates the late competence genes [28–31]. Of the ~100 genes induced, 22 are required for transformation and 6 for fratricide [32,33]. Competence is transient, and its shut off has been shown to involve the late competence gene *dprA* and to proceed through a physical interaction between DprA and ComE~P [34,35] (Fig 1B).

Like other AI based systems, the CSP-based positive loop has been proposed to define a core sensor module here called ComABCDE, through which competence induction is coordinated within the population [36]. The ComABCDE core sensor machinery appears to be in a homeostatic equilibrium that results from the balance of positive and negative inputs during the pre-competence state [17,37], with the former leading to competence shift (Fig 1B and 1C). Once the equilibrium is disrupted by positive inputs, the core sensor is activated in a positive feedback loop that switches the cells into competence. Parameters leading to spontaneous competence shift are not fully understood but appear to be numerous. They include environmental conditions such as initial pH of the medium and the presence of antibiotics, as well as cellular contributions revealed by strain-specific differences in competence development [37–40]. One such difference underlies the question we address in this study. Håvarstein and colleagues succeeded in purifying CSP from the supernatant of a culture of the CP1200 strain, a D39 derivative [15]. This suggested that CSP was able to freely diffuse in the culture medium. In a defined volume, constant CSP production during pre-competence would result in CSP reaching a threshold concentration inducing competence which correlates to a defined and fixed cell density, consistent with a bona fide QS mechanism. In contrast, our preliminary results with the R800 strain, another D39 derivative, showed that the population shifted into competence after a fixed time of growth and independently of cell density [11]. We defined the underlying mechanism as a timing device. Such a timing device has recently been observed for spontaneous competence induction in *Streptococcus thermophilus*, which relies on a specific exported peptide (named ComS) which targets a regulatory system different from that of *S*. *pneumoniae* [41]. In the light of these findings, and to extend our knowledge of cell-to-cell communication during pneumococcal competence development, we have investigated in closer detail the spontaneous shift to competence during planktonic growth of various strains, including representatives of the CP1250 and R800 lineages.

We present genetic evidence demonstrating that spontaneous competence development of a pneumococcal population generally occurs independently of cell density and is linked to the metabolic state of the cells. Hence, we renamed the timing device mechanism underlying pneumococcal competence development as a growth-time dependent (GTD) mechanism. We propose that competence shift is engaged when the sum of stochastic stress perceptions and responses reaches a level that activates the ComABCDE core in certain cells, and so activates the CSP-based feedback loop *via* an autocrine process. The time until the competence shift depends on the lineage genotype and the growth medium used. Competence throughout the population was found to be propagated from the induced fraction of cells through CSP-mediated paracrine activation of non-competent cells. Notably, we show that the neo-synthesized CSP is retained on cells during competence development, providing strong evidence that competence relies on CSP transmission by cell-to-cell contact. Mixed culture experiments performed with a wild-type strain and strains mutant for the ComABCDE module confirmed this mode of CSP transmission. Our experiments also provide evidence that ComD is required for non-competent cells to capture CSP from competent cells.

## Results

### Competence development in commonly used pneumococcal lineages relies on a growth time dependent mechanism

CP1250 (derived from Rx) and R800 (derived from R6) are the two main pneumococcal lineages used to study competence (S1 Fig). However, they behave differently with regard to synchrony of competence development. The CP1250 lineage is reported to develop competence as expected for a *bona fide* QS mechanism, dependent on CSP diffusion [15], whereas competence in the R800 lineage is triggered after a fixed period of exponential cell growth, independently of cell density (for review see: [11,17]). The basis of this difference has not been experimentally determined. As shown in S1 Fig, CP1250 and R800 are derived from the same virulent capsular serotype II parent strain D39 by serial genetic manipulation; notably, the acquisition of a mutation causing loss of mismatch repair in 1959 has resulted in higher mutability, generating the Rx strains from which CP1250 lineage strains have been derived [42,43].

**Fig 2:**
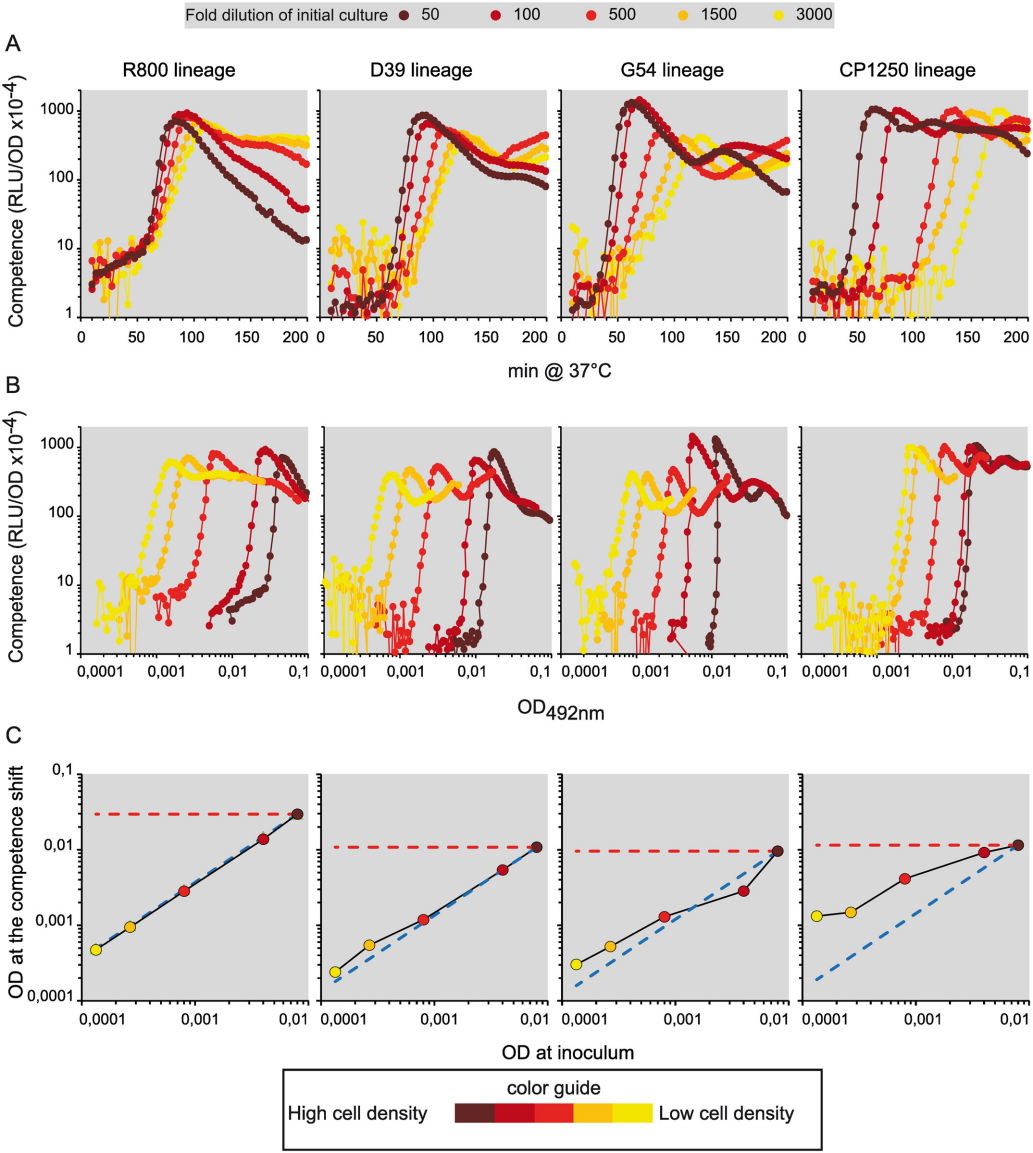
Spontaneous competence development in pneumococci relies on a time-growth dependent mechanism. The 4 strains used R895, TD82, TG55 and TCP1251 harboring the *ssbB*::*luc* fusion to monitor competence development throughout growth have been renamed by the lineage they belong to, R800, D39, G54 and CP1250, respectively. Pre-culture stocks were inoculated at 50, 100, 500, 1500 and 3000 fold dilutions, depicted on the graph as closed dark brown circles, closed brown circles, closed dark orange circles, closed orange circles and closed yellow circles, respectively as described in M&M. RLU and OD readings were recorded as in Fig 1A. Experiments were reproduced at least three times independently, and led to identical results to the one presented. (A) Competence development versus incubation time. (B) Competence development versus cell density. (C) OD at competence shift versus OD of the inoculum. Dashed red line represents a theoretical competence shift relying on a cell density-dependent initiation mechanism (QS). Dashed blue line represents a theoretical competence shift relying on a GTD mechanism. To calculate these two theoretical representations, the fixed OD value considered to set up the theoretical dashed line for each lineage is the one obtained for the highest cell density inoculum. Closed colored circles on the black curve represent data extracted for each strain derivative with color respecting the dilution ratio of inoculum (for data extraction, see M&M).

**Fig 3:**
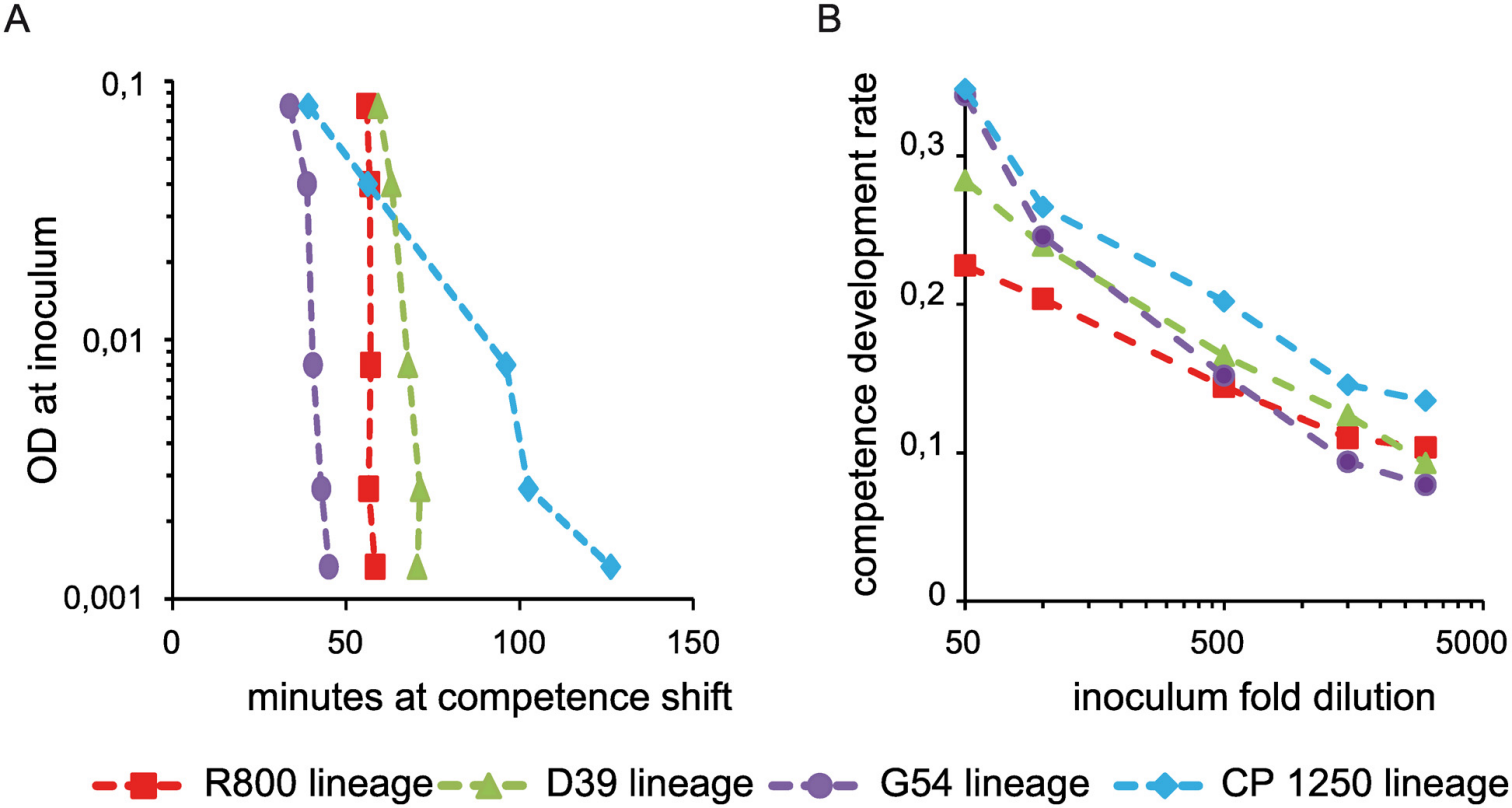
Competence development rate of the cell population depends on cell density. For both graphs: R800 lineage, brown squares, D39 lineage, green triangles, G54 lineage, purple circles and CP1250 lineage, blue diamonds. Experiments were reproduced at least three times and always gave the same profile (see M&M for the mode of calculation). (A) Competence shift time is presented for each strain versus cell density of each inoculum (B) Competence development rate plotted versus fold dilution at each inoculum. Strains used as in Fig 2.

We measured spontaneous competence development for the two strains representing these lineages. To get a broader view, we also analyzed a representative of the original D39 lineage and a recent clinical isolate G54 of the capsular serotype 19F [44]. Introduction into each strain of a *luc* transcriptional fusion to the late *ssbB* competence gene promoter enabled us to monitor competence development in real time during growth [26] (Fig 1A and 1B; see M&M). Competence development was followed as a function of growth time (Fig 2A) or of cell density (Fig 2B). Cells were initially grown for several generations in medium non-permissive for spontaneous competence development. Various quantities of these ‘naive cells’ were then inoculated into medium permissive for spontaneous competence development. The inoculum size used was chosen to ensure detection of competence induction above the photon threshold sensitivity of the luminometer [26] (see M&M). Each strain developed competence in all assays but competence development occurred at different cell densities (Fig 2B). Moreover, apart from CP1250, the competence shift occurred at the same moment during cell growth whatever the size of the inoculum (Fig 2A). We confirmed this behavior by calculating and plotting the competence shift time of each experiment against the OD of each inoculum (Fig 2C). If a population develops competence through QS, the results should fit the red horizontal dashed line corresponding to a fixed OD at the competence shift (arbitrarily taken as the OD reached at the competence shift with the highest density inoculum for each strain). Instead, the results obtained with the R800, D39 and G54 lineages fit a model of competence induction based on a constant growth time, represented in Fig 2C by the blue dashed line. The CP1250 strain appears to exhibit a behavior intermediate between those expected for QS and GTD (growth time-dependent) mechanisms, possibly as a result of its genetic differences from the three other strains (see discussion). Remarkably, the three lineages that trigger competence in a GTD manner do so at a constant time that is specific for each lineage (Figs 2A and 3A).

The competence profiles recorded throughout cell growth show that after the first competence cycle (defined by competence shift to shut off, Fig 1A), subsequent competence cycles correlated with the time of exponential growth in the non-depleted permissive medium (Fig 2A). The four strains exhibit a distinct pattern of these subsequent competence cycles as the growth time elapsed before the first shift to competence is distinctive (Figs 2A and 3A). We also found that this latter property, *i*.*e*. the time of competence shift of a given population, is affected by the nature of the non-permissive growth medium used (S2 Fig). Growth of the R800 lineage in two different non-permissive media before inoculation of the same permissive medium led to competence shifts separated by 42 minutes (bottom graph, S2 Fig). This modulation occurred despite growth rate being the same during the assay (top graph, S2 Fig). Thus, a metabolic memory applies to the cells during subsequent culture in permissive medium. These results show that spontaneous competence coordination in pneumococcal populations depends on growth time and is modulated by both genotype and environmental parameters.

### Competence development relies on a fraction of initiating cells

If each individual cell switches to competence at the same time during growth, competence development within the population should be synchronous and, therefore, should proceed at the same rate independently of inoculum size. However, as is apparent from the plots of Fig 2A, the competence development rate increases with the inoculum size for all strains. Low cell density inoculation leads to a duration of the competence development phase ranging from 46 to 98 minutes much longer than the 25 to 35 minutes observed for the higher cell density inoculation (Fig 2A). This variation was quantified by calculation of the competence development rate for each inoculum size. The competence development rate can vary as much as 3-fold (Fig 3B). This observation strongly argues against autonomous synchronous development of competence by all cells of the population. Rather, this behavior implies that a subpopulation has switched to competence first, resulting in activation of the core sensor and in a high level of CSP production, which then induces the rest of the population through CSP transmission.

### Competence coordination depends on CSP availability

**Fig 4:**
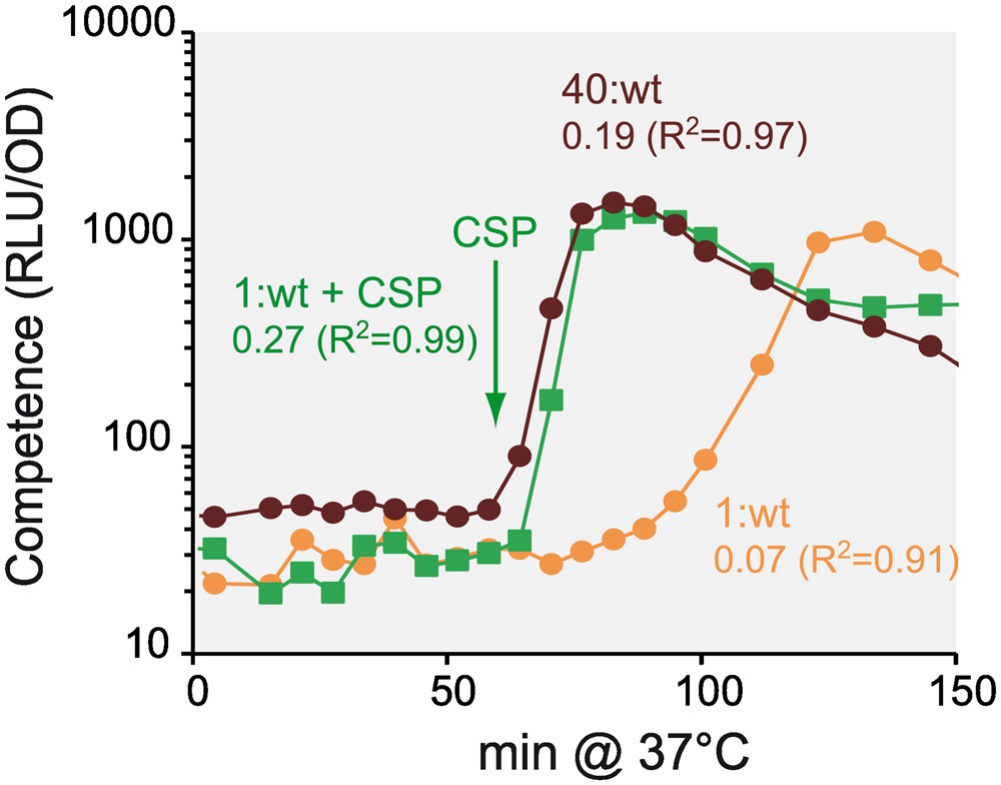
Competence development rate of the cell population is limited by CSP availability. Competence was monitored for the R895 strain inoculated at 50 fold (brown circles, 40: wt) or 2000 fold (orange circles, 1: wt) dilutions of the same pre-culture. The 2000-fold dilution was repeated with the addition of 100 ng/ml of synthetic CSP at the time is pointed by a green arrow (green squares, 1: wt + CSP). RLU and OD readings were recorded in a LucyI luminometer every 6 min (see M.&M). Competence development rate and correlation coefficient R^2^ are presented for each assay.

The two-step scenario for competence development raised the question of the responsiveness of the cells to CSP. We measured this responsiveness by adding synthetic CSP at the time of competence shift to R800 cells grown from a low density inoculum (Fig 4, 1: wt +CSP). As shown previously [15,26], addition of excess CSP to the culture medium provoked instantaneous competence development of the whole population (Fig 4). It proceeded at a higher rate (0.27 RLU.OD^−1^.min^−1^) than when occurring spontaneously (0.07 RLU.OD^−1^.min^−1^) and at an even higher rate in cultures grown from the highest cell density inoculum (Fig 4, 40: wt) (0.19 RLU.OD^−1^.min^−1^). This result clearly showed that CSP concentration is limiting in the low density inoculum culture (Fig 4, 1: wt), while all cells are responsive to CSP. Furthermore, addition of CSP even earlier led to the same result (S3 Fig), showing that all cells are able to respond to CSP at any time during exponential growth. Thus, in addition to the GTD emergence of competence initiator cells, CSP availability is a key factor in the rate of competence development at the cell population level. We next asked how the CSP signal is transmitted from initiator cells to the responders.

### CSP is mainly retained on competent cells

**Fig 5:**
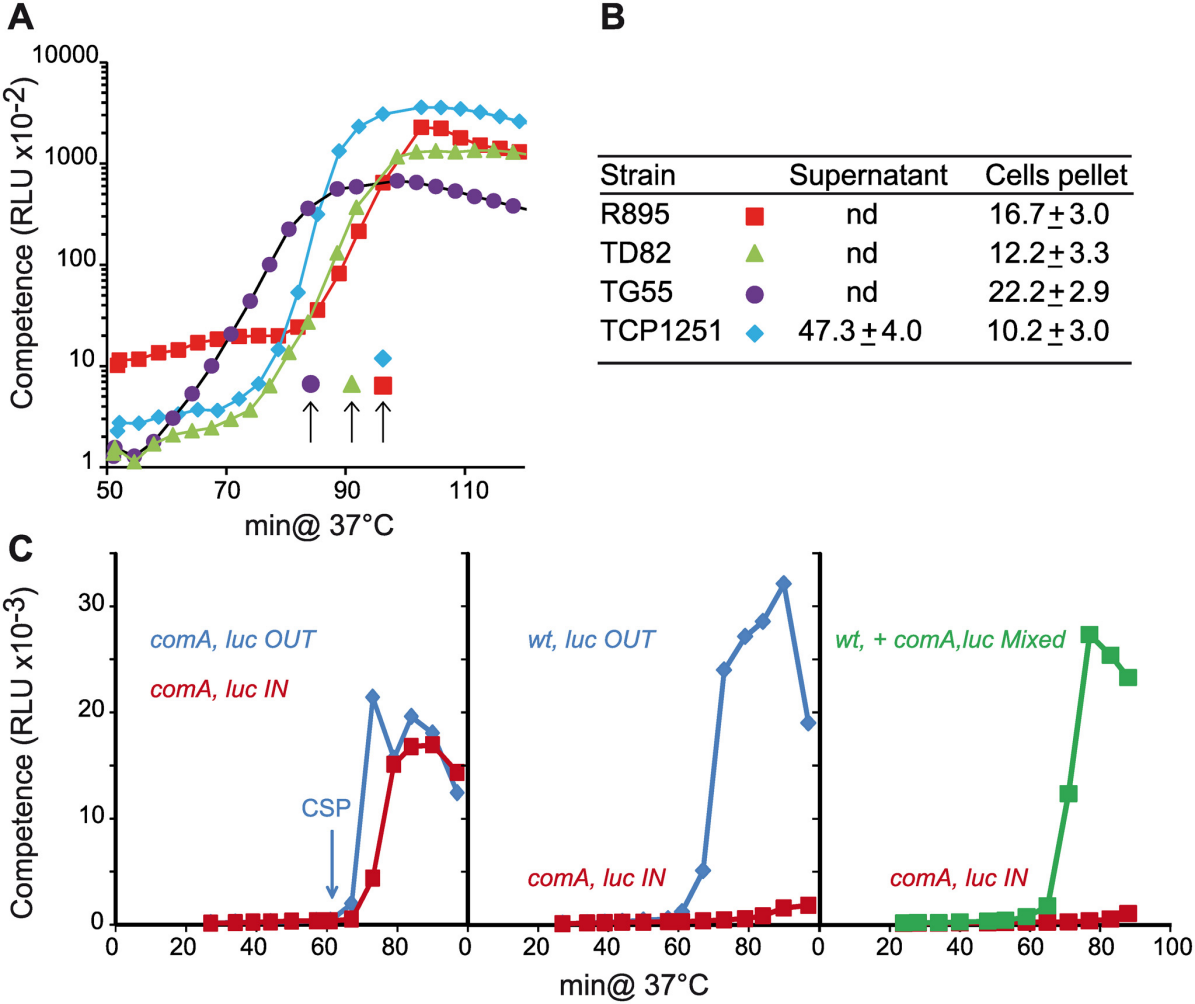
CSP is retained on cells during competence development. (A) Spontaneous competence induction was performed as described in M&M following an inoculation of the cultures by a 20 fold dilution of pre-culture stocks. RLU and OD reading were recorded as in Fig 1. Arrows indicate the times at which aliquots of the cultures were taken to test for the presence of CSP: R800 (R895), brown squares; D39 (TD82), green triangles; G54 (TG55), purple circles; CP1250 (TCP1251), blue diamonds. (B) Estimation of CSP content was conducted as described in M&M; mean and standard deviation values indicate the amount of CSP detected on the cells or in the medium divided by the cell density (ng.ml^−1^.OD^−1^). nd: CSP non detected. (C) Competence of cocultured strains separated by a membrane or not. Blue diamonds, outside compartment, brown squares, inside compartment and green squares mixed culture. Left panel: R1313 (*comA*^*-*^, *luc*) was inoculated in both compartments (OUT, IN) at same cell density. CSP was added in the outside compartment after 61 minutes. Middle panel: R895 (*wt*, *luc OUT*) was inoculated in the outside compartment, and R1313 (*comA*^*-*^, *luc IN*) in the inside compartment. Right panel: R800 was mixed with R1313 in the ratio 3:1 to respect the final volume established for the experiment with compartments separated by the membrane (*wt*, *comA*^*-*^, *luc Mixed*). For the separated experiment, R800 is inoculated in the outside compartment and R1313 (*comA*^*-*^, *luc IN*) in the inside compartment. Each experiment was repeated at least twice.

Purification of CSP from a CP1250 lineage culture supernatant [15] had suggested that cells communicate through free diffusion of CSP in the medium. However, in the first attempt to isolate pneumococcal CSP, its activity was obtained not from the culture supernatant but from pelleted heat-killed R6 cells [40], implying that most CSP remained bound to the cell envelope. We re-investigated CSP distribution during spontaneous competence development in the four above-mentioned lineages. Media were inoculated with naive cells at high density to maximize synchronization of competence development, and cells were collected by centrifugation at the time of maximum competence. The supernatants and heat-treated cell pellets were assayed for the presence of CSP, using the luciferase activity of a *comA*^*-*^ strain harboring the *ssbB*::*luc* transcriptional fusion as a reporter of ability to induce competence (Fig 5A). Synthetic CSP was used as a standard for these quantifications. In all cases, CSP was found in the cell pellet, and at similar concentrations (Fig 5B). CSP was detected in the supernatant only in the case of the CP1250 strain, whose total CSP production was 2–5-fold higher than that of the other strains. Thus, apart from strain CP1250, pneumococcal cells retain CSP on their surface without releasing it efficiently into the medium during competence development. This feature strongly suggests a basis for the difference in competence development between the CP1250 lineage and the others.

To test if cells retain CSP during competence development, we set up co-culture experiments with strains separated by a porous membrane with a 50 kD cut off (see M&M). The porosity of a 50 kD membrane should allow diffusion of the 2.2 kD CSP without permitting cell contact between the two compartments. First, we validated the ability of CSP to diffuse through the membrane. To this end, we inoculated the *comA*^−^ mutants reporting competence development in both compartments at the same cell density and added CSP after 1 hour of growth in the *OUT* compartment (Fig 5C, left panel, *comA*^*-*^, *luc OUT*). Both cell populations (*comA*^*-*^ *luc OUT*; *comA*^*-*^ *luc IN*) were found to develop competence concomitantly (Fig 5C, left panel), demonstrating that the membrane does not block CSP diffusion. Next, we performed the same experiment by inoculating the *OUT* compartment with wild-type cells and the IN compartment with *comA*^*-*^ cells, with both strains harboring the *luc* reporter (Fig 5C middle panel, *wt*, *luc OUT; comA*^−^, *luc IN*). In these conditions, wild type cells develop competence naturally but *comA*^*-*^ cells do not develop competence concomitantly, suggesting that CSP has been retained by the wild type cells. Finally, we repeated the experiment by mixing a wild type strain that does not report competence by luciferase expression with the *comA*^−^ *luc* strain in the same compartment, and compared competence induction with the experiment performed with these two strains cultivated into the two compartments device (Fig 5C, right panel). Competence development of *comA*^*-*^ cells induced by wild-type cells was observed only in the mixed cells experiment. Altogether, these experiments show that CSP is preferentially retained by the producing cells and that its propagation throughout the population is favored by cell contact.

### Competence propagation between the wild type cells is blocked by an excess of *comA*^*-*^ cells

Retention of CSP by the cell indicates that its transmission could occur by random collision. At high cell density, competence propagation would thus be favored leading to a high rate of competence development. We suggest that a subpopulation of cells present at the inoculum of the culture is at the origin of competence initiation and propagation through the population (Figs 2 and 3B). We tested this prediction by measuring competence development in wild type R800 carrying the *ssbB*::*luc* fusion gene (competence reporter cells) co-cultured with an excess of isogenic non-reporter cells.

**Fig 6:**
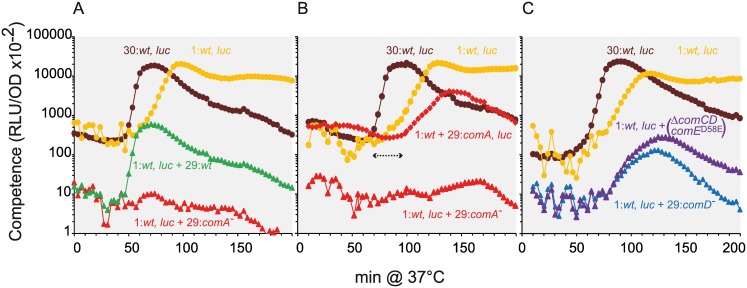
ComD is involved in CSP retention on receptor cells. (A) Competence development of wild-type cells is blocked by an excess of *comA*^*-*^ cells. Competence has been recorded as described in M&M. For single or mixed cultures, strains genotypes with relative cell ratios at the inoculum are indicated as follow: orange circles, (1: *wt*, *luc*) for a 1500 fold dilution of the R895 strain alone; brown circles, (30: *wt*, *luc*) for a 50 fold dilution of R895 strain alone; green triangles, (1: *wt*, *luc* + 29: *wt*) for a 1500 dilution of R895 strain mixed with a 50 fold dilution of R800-wild type strain; red triangles, (1: *wt*, *luc* + 29: *comA*^*-*^) for a 1500 dilution of R895 strain mixed with a 50 fold dilution of R1625 strain (*comA*^−^). Note that in mixed cell populations, i.e. R895/R800 and R895/R1625, the inoculum of the competence reporting strain being 1/30^th^, the RLU.OD^−1^ value recorded during the pre-competence period is ~30 fold lower. (B) *comA*^*-*^ cells recover CSP produced by the wild type initiator cells. The experiments were repeated as in panel A and represented similarly. In addition, red diamonds (1: *wt* + 29: *comA*^*-*^,*luc*) represent a mixed culture of the R800-wild type strain with 30 fold more concentrated *com*A^−^ R1313 strain that reports competence. (C) ComD is involved in CSP capture on receptor cells. Experiments were performed as described in panel A. Blue triangles (1: *wt*, *luc* + 29: *comD*^*-*^) and purple triangles (1: *wt*, *luc* + 29: Δ*comCD comE*^D58E^) represent competence measurements recorded for mixed cultures of the wild-type R895 strain with the *comD*^−^ R1745 strain or theΔ*comCD comE*^D58E^ R2977 strain, respectively.

The wild-type reporter strain inoculated with a 30 fold excess of the wild-type non-reporter strain (1:*wt*, *luc* + 29:*wt*) developed competence at a 3-fold higher rate than when grown alone at the same low density (1:*wt*, *luc*), *i*.*e*. the competence development rate rose from 0.048 to 0.148 RLU.OD^−1^.min^−1^ (Fig 6A). This higher rate was equivalent to the rate measured with the wild-type reporter strain inoculated at high density (30: *wt*, *luc*; 0.124 RLU.OD^−1^.min^−1^; Fig 6A). Such a result was expected since, the reporter gene apart, all cells in the culture are of identical genotype. Both populations contribute to appearance of the cell fraction initiating competence and have the same CSP production and sensing capability. In the mixed culture, the wild type non reporting cells act as helper cells facilitating both production and transmission of CSP between reporter cells. However, the equivalent mixed culture experiment performed with a *comA*^−^ non-reporter strain (1: *wt*, *luc* + 29: *comA*^*-*^, Fig 6A), prevented competence development of the wild-type reporter strain. The *comA*^−^ non reporter cells, which are responsive to CSP but unable to export it, act as cheater cells by blocking CSP transmission. Because the *comA*^−^ cells heavily outnumber the wild type reporter cells, most of reporter cell collisions are with *comA*^−^ cells. Consequently, *comA*^−^ non reporter cells reduce the frequency of wild type reporter cell collision thereby diminishing competence propagation among this minority. The *comA*^−^ cells can be fully induced to competence if sufficient CSP is available (S4 Fig). Therefore, *comA*^*-*^ reporter cells present in excess with wild type non reporter cells should switch to competence, but CSP transmission will occur only upon contact with the initiating cells. As shown in Fig 6B, in the same mixed population but with only the *comA*^*-*^ cell reporting competence (1: *wt* + 29: *comA*, *luc*), a 30 minute delay in competence shift and a damping of competence development were observed. Details of CSP production by the wild type cells are presented in S4 Fig.

These results strongly support the notion that competence initiation relies on a distinct cell fraction. Moreover, when CSP is transmitted to a *comA*^*-*^ receiver cell, it appears to be no longer accessible to the quiescent and responsive neighboring wild type cells.

### ComD acts as a CSP captor during competence propagation

Previous experiments showed that *comA*^*-*^ cells, while reactive to CSP and to wild-type competence initiator cells, captured CSP within the population and impeded the propagation of competence. ComD is the transmembrane sensor which is presumed to transmit the CSP signal by phosphorylating its ComE partner [27,45,46]. To determine whether ComD is involved in CSP capture and retention, we used mixed culture experiments to examine how non-reporter *comD*^*-*^ cells, which are unable to react to CSP, affect competence development of wild-type reporter cells. If ComD is the major CSP captor, then in the mixed population the *comD*^*-*^ cell would act as deaf-mute cells since the core sensor is inactivated. The competence development rate of the mixed culture (1: *wt*, *luc* + 29: *comD*; Fig 6C) was found to be slightly reduced (about 1, 5 fold) in comparison to that of the pure wild type reporter culture (1: *wt*, *luc*; Fig 6C). This result sharply contrasts with the inhibitory effect of the *comA*^*-*^ non-reporter strain on competence development of the wild-type strain (Fig 6A). Thus ComD appears to be a central element in the capture and retention of CSP by receiver cells. To test whether ComD itself or another protein of the competence regulon under its control (through ComE activation) is responsible for CSP retention, we repeated the mixed culture experiment using a *comE*^D58E^ strain, which renders the competence regulon constitutive [27] but containing *comC*^*-*^ *comD*^*-*^ mutations that prevent CSP export. The competence development rate of the mixed culture was similar to that obtained with the *comD*^−^ strain. Therefore, it is unlikely that a product of the competence regulon other than ComD contributes significantly to CSP retention on receiver cells. Unproductive cell collision with the large excess of *comD*^*-*^ cells may passively contribute to reduction of the competence development rate of the wild type reporter strain, thus producing a buffering effect of about 1.5 fold.

### Pneumococcal lineages are distinguishable by their capability to generate a cell fraction initiating competence

The four pneumococcal lineages analyzed display distinct pre-competent time periods when grown under identical conditions (Fig 3A). Moreover, the pre-competent time period of CP1250 varies as a function of the density of the inoculum, in contrast to the three other lineages for which the pre-competence time period is nearly constant (Fig 3A). This variation indicates that a property of the mechanism driving competence development is modified in CP1250. This modification concerns the pre-competence period, during which a fraction of cells has switched stochastically to autocrine activation of CSP overexpression. We propose that both the number of stress-induced cells at competence shift and the cell density in the culture determine the shift and the rate of propagation of competence throughout the population. By diluting the inoculum of CP1250, the threshold value of one or both of these two parameters is not reached at the same pre-competence time period of the previous and denser inoculum, and competence propagation thus does not occur at this time. Further exponential growth will generate new stress-induced competent cells, which could attain the two threshold values needed for triggering competence propagation later during the culture, giving a larger pre-competence time period. This also means that the distinct categories of cells that will switch individually during the pre-competence period are already present in the initial inoculum. This scenario is strongly supported by the experiment presented in the S5A Fig, where the range of cell density at the inoculum was extended to higher cell density than previously used in Fig 2. The CP1250 lineage presents a constant pre-competence time period for the inoculum above OD 0.01, while after this point, progressively higher pre-competence time periods are observed as the density of inoculums decreases. Thus, the pre-competence period is defined by the amount and concentration of stress-induced cells that have switched into CSP overexpression by an autocrine mode leading to the competent shift. The CP1250 lineage is thus convenient to investigate this hypothesis. Indeed, the use of the *luc* reporter gene remains in the range of sensitivity of the luminometer detector since the constant pre-competent time period is lost below 0.01 OD. We repeated the assays below and above the threshold cell density several time each to collect several pre-competence time periods. For the 12 assays performed at high cell density, we observed a reproducible pre-competence time period with a mean value of 60 minutes of growth ranging from time 59 to 64 minutes (S5B Fig, top panel). But for the 36 assays conducted with a 3 fold lower cell density, the pre-competence time periods ranged from 64 minutes to 91 minutes of growth with 27 cultures developing competence in a narrower window of time from 76 minutes and 84 minutes (S5B Fig, bottom panel).

These results support the existence of a category of cell present at the inoculum determining the pre-competence time period, which depends on the number and proportion of initiating cells at the higher cell density. By multiplying the number of assays at lower cell density inoculum, we have conserved the number of initiating cells but these are randomly scattered in the different assays which in most cases results in random loss of either the number or the ratio of these cells required maintaining the pre-competence time period of the higher cell inoculum. But some assays at lower cell density have maintained both parameters by random distribution to initiate competence with a pre-competence time period comparable to the high cell density assays (S5B Fig). The large majority of the assays at low cell density inoculum have a significant increase of the pre-competence time period spanning a wide range of time (27 minutes). This wide range corresponds to the time necessary to produce the missing number and proportion of cells able to initiate competence in an autocrine mode. This hypothesis also explains the results observe in Figs 2A and 3A with a similar pre-competent time period for two different cell densities of the CP1250 lineage below the threshold cell density.

## Discussion

### Pneumococcal competence is propagated *via* a cell-contact sensing mechanism

**Fig 7:**
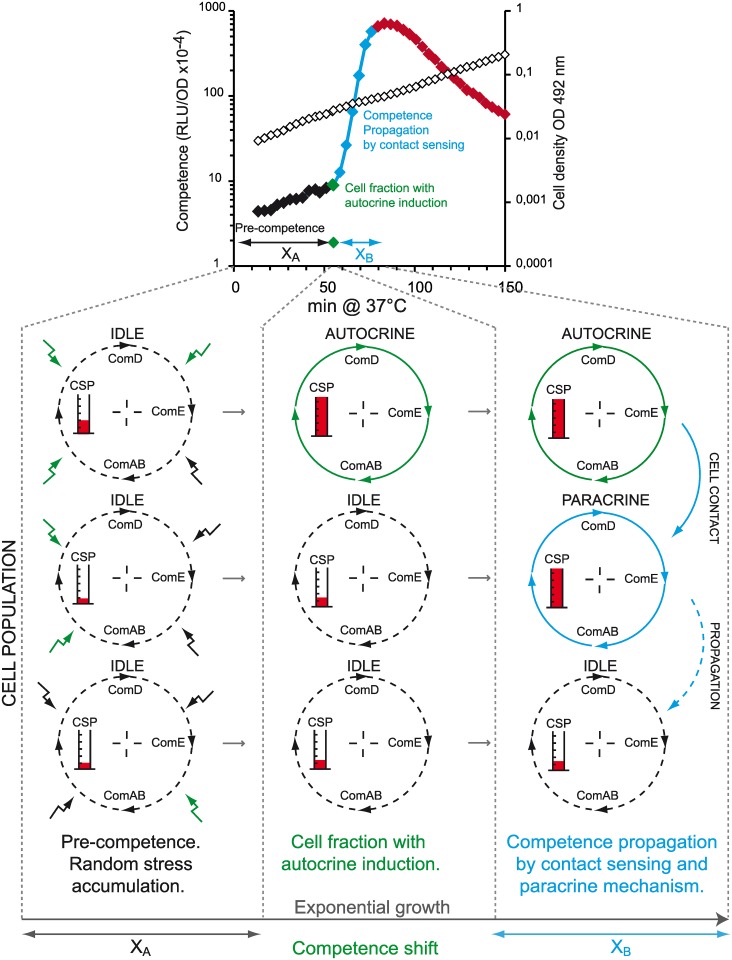
Pneumococcal competence is induced by a cell fraction and propagated by cell contact throughout the population. On the classical competence profile of a growing planktonic cell population described in Fig 1A, the X_A_ and X_B_ values correspond to the duration of the pre-competence and the competence development periods respectively, separated by competence shift. On the bottom part of the graph, each column represents how the ComABCDE core sensor module (Fig 1C) evolves in individual cells during these 3 distinct stages. During the pre-competence period (left column), distinct and various stresses (lightning arrows) are sensed by individual cells either as positive input (green) or negative input (black) modifying the state of their core sensor accordingly (symbolized by measuring cylinder cartoon). The core sensor could be turned on in some cells *via* an autocrine mode in response to competence inducing stresses. Upon reaching a threshold value at X_A_ this subpopulation triggers competence propagation throughout the population at the shift point (medium column). This propagation proceeds by CSP transmission *via* cell-to-cell contact during the X_B_ period (right column). During competence propagation, the receptor cells switch into competence by turning on their ComABCDE core sensor *via* a paracrine mode (core sensor colored in blue).

This study provides evidence that population-wide pneumococcal competence development in liquid cultures is a two-step process: a fraction of the cells initially switches to CSP over-expression; these cells then induce competence in the whole population. We propose that this second step occurs by transmission of CSP between cells via random cell-to-cell collision. The competence shift point (Fig 1A) marking the boundary between these two steps generally occurs after a constant time of growth (termed X_A_; Fig 7), whatever the density of the culture inoculum (Figs 2 and 3A). The switching of a fraction of cells to competence during the X_A_ period (Figs 1A and 7) results from auto-activation of the CSP-based positive feedback loop in those cells. CSP would then decorate the producing cells, potentially rendering it accessible to neighboring quiescent cells by random collision (Fig 1C). Conversely, the propagation of competence throughout the population lasts a longer period of time (termed X_B_; Fig 7) that is inversely proportional to the cell density of the inoculum (Fig 3B). It relies on a paracrine CSP transmission mode mediated by direct contact between the donor and receiver cells, as CSP does not generally diffuse easily in the medium during competence development (Figs 5 and 6). This cell-contact sensing model is clearly different from known models of sensing [6–8]. The ComAB ABC transporter delivers CSP outside of the cell, but nothing is known about how CSP reaches its ComD target. In light of this present study supporting the notion that the CSP is transmitted by cell contact, it becomes important to understand how such a transmission operates. It seems that, once bound to ComD, the CSP is not released during the competence development period (Fig 6). This suggests that only CSP freshly exported by ComAB is available for a ComD unbound by CSP located either on the producer cells or on the receiver cells.

### Stress-induced noise creates the initiator sub-population

Ours results are consistent with the idea that spontaneous competence development at the population level is a suite of events that begin by the autocrine competence activation of some individuals that may propagate competence to the neighborhood. In this model, the core sensor ComABCDE module is central to both steps of pneumococcal competence development. During the X_A_ period, this module registers stresses at individual cell level. We propose that core sensor of each cell is either in an idle or activating mode (Fig 7). According to such a classical bistability model [47], a cell sub-population first responds most acutely to stimuli, and second compels the other cells to follow this change in order to drive a new genetic program (Figs 2A–2C and 3). Applied to pneumococcal cultures, competence is an answer to stresses, acting as an alarm by physically contacting the closest cells to spread competence during the X_B_ period (Fig 7, S6 Fig). For several organisms, the role of stress-induced “noise” in initiation of different gene expression routes in a clonal population is considered to be the promotion of varied physiological states [47–51]. Competence may provide a way for a cell population to survive environmental threats such as antibiotics or the host immune system by acquisition of new genetic traits via transformation [24,25]. However, the benefits of a switch to competence could be offset by the possible incorporation of deleterious genes or mutations by transformation and also by the physiological change of the cell that accompanies competence, so that in order to realise the benefit it has to be shut down again quickly. It is then not surprising that competence induction is controlled according to growth conditions [16,38,39] (S2 Fig) and varies also as a function of genotype (Fig 3A). The X_A_ value of the pre-competence period corresponds to the time taken to accumulate physiological signals that eventually push the *comABCDE* loop and hence CSP production over a threshold value. Thus, ComABCDE is a multi-sensor that can be tuned up or down by integrating stress signals (Figs 1 and 7). Each step in ComABCDE core sensor influences the idling balance that determines the ComE/ComE~P ratio [16]. Any factor affecting the ComABCDE core by positive input, such as initial alkaline pH of the medium, antibiotics or DNA damaging agents, may stochastically favor a shift to competence initiation [11,37]. Some signals, such as misfolded proteins, stalled replication forks or proteins like the serine threonine kinase StkP, act to stimulate the core sensor loop [52–55], while the two component regulator CiaRH represses the core sensor by its control of csRNA and HtrA proteinase expression [28,55–57]. Variability of the X_A_ period presumably reflects response to these multiple inputs. The sensitivity of the core sensor to external and internal signals can be modulated, by many direct or indirect effects (see also S1 Text). Some responses could amount to irreversible physiological changes that commit a cell to competence well before core sensor activation boosts the CSP level (S2 Fig). Competence bistability model should be tested in further experiments. Focus on the spontaneous competence development at single cell level should be an interesting perspective to explore the stochasticity of the autocrine competence development.

### CSP is retained on the cells

We have demonstrated here that neo-synthesized CSP is retained on competent cells of four distinct pneumococcal lineages. This is a general feature of pneumococci and is central to the concept of a cell-contact sensing mechanism. The steepness of the competence development curves is directly proportional to the cell density at competence shift, as expected if determined by the probability of cell collision (Figs 2 and 7).

A notable difference between the CP1250 lineage and the three others analyzed is the presence of a large amount of CSP in the CP1250 culture supernatant at the X_B_ period. Nevertheless, competent CP1250 cells retain one fifth of the CSP they produce, amounting to a number of CSP molecules similar to those produced by the three other lineages. CP1250 is known to express the early *com*CDE and *com*X competence genes for longer than R800 [27]. This may lead to greater CSP production before competence shut-off, and explain the release of excess CSP into the medium. But even with this large excess of CSP in the medium, competence coordination in CP1250 cultures does not match that predicted by the QS model (Fig 2B and 2C). This suggests that the cell-contact sensing mechanism plays an important and dominant role even in this lineage.

CSP retention on the cell surface (Fig 5) could be explained in part by its ability to adopt an amphiphilic helical configuration [58]. Upon export via ComAB, CSP has a hydrophobic face and could be embedded in the lipid membrane. Membrane attachment of CSP is consistent with the observation that the membrane-anchored protease, HtrA, modulates its abundance by direct degradation [57]. In addition, our results suggest that CSP is not released from *comA*^*-*^ mutant cells and is retained by ComD (Fig 6). The relatively large number of ComD molecules present before competence induction, 1 500 per cell [27], would favor interception of CSP. In addition, ComD reaches 39 000 molecules per competent cell, which would therefore contribute to avoid free CSP diffusion. Cell contact appears central for CSP transmission throughout the cell population cultivated under planktonic conditions. Nothing is known about how this event occurs. One possibility would be through the co-aggregation of cells during the growth. It has been shown under artificial acidic conditions of growth that competent cells present a clumping capacity with non competent cells [59,60], but this study described late events in competence development involving fratricide and DNA release from the lysed cells. The pneumococcus usually lives in a biofilm in the human nasopharynx, a mode of growth resulting in a higher transformation efficiency than during septic infection [61]. CSP retention on cells and transfer by cell contact in a biofilm would favor competence propagation to the close neighbors of the induced cells and thus to their clonal siblings.

### ComD is responsible for the majority of CSP retention on the cell surface

Interaction between ComD and CSP is central for physical retention of CSP on producing cells, preventing free CSP diffusion in the medium during the pre-competence and competence development periods (Figs 5 and 6C). The other CSP fraction available is the one freshly released by the ComAB transporter that is not yet captured by the resident ComD and may promote competence propagation by allowing quiescent contacting cells to acquire CSP. ComD-CSP interaction mediates ComE-dependent activation of competence genes needed for genetic transformation and fratricide [17]. An analogous case is that of the CbpD fratricin effector, which remains anchored to the teichoic acid of the cell wall and whose lytic action against neighbors is mediated by cell contact [62]. This emphasizes the importance of a basal level of comD expression in non-competent cells. It would allow non-competent cells to survive fratricide by developing competence upon CSP transmission via cell-contact and by overexpressing the immunity factor [63–66].

Conversely, this will eliminate cells that no longer express ComD. In addition, pneumococci and close relatives have evolved several specific pairs of CSP-ComD alleles [67,68]. Therefore, competence development in the initiator fraction of pneumococcal cells would also promote lysis of cells with a different CSP-ComD pair during the propagation step (S6 Fig). Such killing of siblings during competence might provide a source of exogenous DNA for the transformation process [17], which could favor genome plasticity and/or repair [13]. In addition, propagation of competence from cell to cell provides an efficient way to maintain an active CSP-based positive feedback loop in all cells of the progeny.

### Concluding remarks

Within the bacterial kingdom, the ability to convert an entire population to the competent state appears to be restricted to pneumococci and close relatives. This may provide particular properties to the whole population, such as the consequences of fratricide (as discussed above) or other properties provided by the many competence genes of unknown function. The cell-contact sensing mechanism driving this collective behavior is novel amongst the AI-based sensing mechanisms characterized so far. Some of its key features apply also to spontaneous competence development in *Streptococcus thermophilus*. Like pneumococcal competence, it relies on an exported peptide, named ComS, and is induced in a GTD manner independently of cell density [41]. ComS appears to be retained on the cell surface but, by contrast with the CSP, is re-imported into the cell where it mediates transcriptional activation of competence genes. Interestingly, ComS might also be sensed by neighboring cells. This indicates that competence of *S*. *thermophilus* could also propagate by cell-contact sensing as revealed here for pneumococcal competence. Thus, transmission of AI signals by cell-cell contact may be far more general than the population-wide propagation of pneumococcal competence revealed by our results. In their natural environment, pneumococci can be found growing in biofilms or dispersed in liquid. These two distinct lifestyles may determine how competence is propagated in different niches during colonization and infection. Understanding the differences in competence transmission in these different modes of growth, and whether the cell-contact sensing mechanism is used in both cases, should provide insight into the importance of competence to pneumococci in different niches.

## Materials and Methods

### Bacterial strains, culture and transformation conditions

*Streptococcus pneumoniae* strains are described in S1 Table. To observe spontaneous competence development the following procedure is conduced. Except where noted, stock cultures were grown at 37°C to 0.2 OD 550 nm in competence non-permissive medium Casamino Acid Tryptone (CAT) adjusted with HCl to pH 6.8. The cells were washed by centrifugation, suspended at 0.4 OD 550nm in C+Y medium [69] containing 15% glycerol and frozen at -80°C. Inoculation of C +Y medium pH 7.9 (permissive medium) with these stock cells allows spontaneous competence development. To monitor competence, all strains contained the transcriptional fusions with the *luc* firefly luciferase gene under the control of a competence regulated promoter (S1 Table). Cultures were started by diluting various volumes of stocked cells and a 300μL volume of C+Y medium with luciferin, with the inoculum used for monitoring *luc* expression in clear bottomed wells of a 96-well white NBS micro plate (Corning) [26]. Relative luminescence units (RLU) and OD values were recorded throughout incubation at 37°C in a LucyI (Anthos) or a Varioskan Flash (Thermo 399 Electron Corporation) luminometer. All experiments were repeated at least 3 times. To detect the first round of competence development in the population, all assays, especially for low cell density inoculate, were designated to record RLU values from the cell population above the threshold photon detection [26]. As the sensitivity of the Varioskan Flash and LucyI are limited in OD detection for very low inoculate, a mathematical calculation to obtain the approximate OD value under the threshold detection was carried out, using the OD know by the dilution of the pre-cultured cell at time zero and the values detected above the threshold sensitivity of the Varioskan Flash and LucyI to extract the exponential parameters of the growth culture. Calculation of the competence shift time and competence development rate: the X coordinate values corresponding to competence shift time have been estimated as followed. Exponential regression was calculated by extracting at least 5 consecutive measurements in the curve portion of the competence development phase for each competence development portion curve when reporting RLU.OD^−1^ (y ordered) expressed against time (x-axis). However exponential regression was calculated by extracting 3 consecutives measurements for two experiments in Fig 4 since RLU and OD was recorded every 6 min. The correlation coefficient R^2^ for each exponential regression calculation was found to be between 0.91 and 0.99. The competence development rate corresponds to the r parameter extracted from the exponential regression equation a*exp(r*t). Each exponential regression allows calculation of an X value (time value for competence shift) which corresponds to the intersection between regression function of competence development rate with the mean value before competence shift.

CSP-induced transformation was performed as described previously [70] using pre-competent cells treated at 37°C for 10 min with synthetic CSP1 (100 ng ml-1). After addition of transforming DNA, cells were incubated for 20 min at 30°C. Transformants were selected by plating on CAT-agar supplemented with 4% horse blood (10ml), incubating for 2 hours at 37°C for phenotypic expression, and overlayed with 10 ml CAT-agar containing the appropriate antibiotic as followed: chloramphenicol (4.5 mg ml^−1^), kanamycin (250 mg ml^−1^).

#### Sampling for CSP dosage

Strains were grown in CAT medium pH 6,8 to 0,15–0,3 OD_550_ nm, concentrated to OD 0,4 and store at -80°C. Inocula were diluted to OD 0,01 in micro plates (300 μl cells with 0.17 mg.ml^−1^ luciferin) and incubated at 37°C. 3.6 ml sampling (12 wells) were recovered for each assay on micro plate. Cells were removed by centrifugation for 10 minutes at 14,000 rpm (in an Eppendorf 5417C) micro-centrifuge. The supernatant was stored at -80°C. Cells pellets were suspended in 100 μl C+Y and incubated for 10 minutes at 60°C, then stored at -80°C.

CSP measurement: R1313 (*comA*^*-*^*; ssbB*::*luc*) cells were grown in C+Y pH7.9 to 0, 15 OD _492_ nm and 100μl aliquots were transferred to a clear bottomed NBS 96-well white micro plate containing 0.26 mg.ml^−1^ luciferin per well. CSP in C+Y was added at various concentrations to generate a standard calibration curve of competence induction by integrating the competence response curve. Samples of supernatant and cell pellets from culture at the peak of competence induction were assayed in the same way. Mean values and standard deviations were obtained from at least 3 measurements and are expressed as ng.ml^−1^.OD^−^1.

### Spontaneous competence development in co-cultures separate by a porous membrane

Two pre-cultured strains (chosen between R895, R1313 and R800) grown in non-permissive medium (see above) were inoculated at OD 0.0067 in C+Y pH 7.9 permissive medium with the same cell density in two compartment separated by a porous membrane with a molecular cut off of 50 kD (Float-A-Lyser; Spectrumlabs). The outside and the inside compartment had a final volume of 15 ml and 5 ml respectively which allows a higher sensitivity of the inner compartment to molecule diffusion from the outer compartment. As a result, the cells are at the same density in both compartments but with a final ratio of 3 to 1. Measurements of competence were conducted in the Varioskan Flash luminometer by taking 100μl aliquots from each compartment every 6 minutes and adding these to microplates containing luciferin for RLU reading. The experiment with CSP addition was realized to reach 100ng.ml^−1^ in the outer compartment.

#### Spontaneous competence development in strain-mixing experiments

Two pre-cultured strains grown in non-permissive medium (see above) were inoculated at a 1:29 ratio except as indicated in the figure experiment in C+Y pH 7.9 permissive medium with luciferin. The minority strain was added at a 1500 fold dilution and mixed with the majority strain to reach the equivalent of a 50 fold dilution. Spontaneous competence was followed as described above.

## Supporting Information

S1 Fig Pneumococcal lineages and relationships. Years indicate the first published mention of each strain. Red rectangles/boxes indicate virulent clinical isolates and blue boxes the derivatives used in this study The R36A rough strain was isolated by Avery and coworkers in 1944. It was obtained after 36 serial passages on a rabbit serum raised against a smooth pneumococcal type II strain. SIII-1 is an R36A transformant that kept the rough phenotype after exposure to DNA containing the serotype III capsule locus, and was subsequently renamed Clone 3 [71]. R6 is a single clone of the R36A strain, kept for its natural transformation capacity as measured in the growth medium used by Ottolenghi and Hotchkiss [72]. R800 is a R6 transformant which has acquired a suppressor phenotype from Cl3 chromosomal DNA restoring normal colony size when selected for aminopterin resistance. The SIII-N strain is an R36A transformant that has acquired the type III serotype of the A66 type III strain. Rx is a rough spontaneous mutant, which has also lost mismatch repair ability. CP1250 was derived from CP1200 by serial transformations and mutagenesis, resulting in acquisition of resistance to streptomycin and of inability to ferment maltose and β-galactosidase inactivation. TD82 (D39 lineage) and TG55 (G54 lineage) result from transformation of the D39S and G54 clinical isolates, respectively, to abrogate capsule synthesis, followed by introduction of the *ssbB*::*luc* transcription reporter gene. R895 (R800 lineage) and TCP1251 (CP1250 lineage) are direct derivatives of R800 and CP1250, respectively.
(EPS)

S2 Fig Growth time competence induction depends on cell metabolic memory. Samples of R825 precultures growing exponentially in either CAT medium or C+Y adjusted to pH 6.8, to impede competence development, were collected, washed and added to C+Y adjusted to pH 7.9 to permit spontaneous competence development. OD_492_ (upper panel) and RLU/OD_492_ (lower panel) measurements of cultures started by 50-fold or 500-fold dilution of pre-cultures, as shown by the colored symbols. All cultures grew at the same rate (upper panel). RLU and OD_492_ readings were recorded in a LucyI Anthos luminometer at 15-min intervals. In all cultures competence development proceeded according to a GTD mechanism, although the X_A_ period prolonged by 42 minutes in cultures started from an inoculum prepared in CAT in comparison to that from C+Y. (lower panel).
(EPS)

S3 Fig The cell population can respond to synthetic CSP at any time of the exponential growth. Competence was monitored as described in Fig 4. On each graph, the blue diamond curve represents the spontaneous competence profile of cells from a 2000-fold dilution of cells grown in C+Y pH 6.8 (M&M). The second curve is the competence profile of such cells to which synthetic CSP (100ng.ml^−1^) has been added at 15, 27, 39, 52, 64 and 76 minutes after dilution, RLU and OD_492_ were at 6-minute intervals.
(EPS)

S4 Fig Estimation of CSP captured by the *comA*^−^ cell population in mixed cultures. To estimate the number of CSP molecules produced by a minority of wild type cells and captured by *comA*^−^ cells in the co-culture experiments presented in Fig 6B, we performed a parallel experiment in which known amounts of CSP (expressed in ng.ml^−1^) were added to *comA*^−^ cells harboring the competence-reporter module, *ssbB*::*luc* (“*com*A, *luc”*). Using this calibration (left panel), we estimated that an average of 1500 CSP molecules are captured by each *comA*^−^ cell in mixed culture inoculated with a 30-fold minority of wild type cells (1: *wt* + 29: *comA*, *luc*; right panel). The experiment was performed by mixing a 1500 fold dilution of a pre-culture of the *comA*^*-*^ strain (R1625) with a 50 fold dilution of an equally concentrated pre-culture of the competence *comA*^−^, *luc* reporter strain (R1313). The culture was divided into 12 aliquots, and CSP (arrow) was added to each at the concentrations shown after a growth time that correspond to the X_A_ time observed for the (1: *wt* + 29: *comA*,luc), right panel. The calibration curve was obtained by the integrations of each competence response to CSP. The number of CSP molecules sensed per *comA*^−^ cell was calculated by dividing the quantity of CSP produced by the wt in the experiment (1: *wt* + 29: *comA*, luc) by the number of *comA*^−^ cells in the mixture.
(EPS)

S5 Fig X_A_ value of the pre-competence period of R800 and CP12500 strains as a function of inoculums size. (A) Pre-cultures were inoculated by 20, 30, 40, 50, 100, 500 and 1500-fold dilution (see M&M). The X_A_ value of the pre-competence period is presented for each inoculum size (see M&M): R800 lineage, brown squares; CP1250 lineage, blue diamonds. X_A_ values were estimated as described in Fig 3A. (B) TCP1251 pre-culture stocks were inoculated at 30 and 90 fold dilutions in C+Y pH 7.9 permissive medium (see M&M) and repeated 12 times and 36 times respectively. Top panel represent competence development of the 12 experiments inoculated at high cell density and the lower panel represent competence development of the 36 experiments inoculated at low cell density. The red horizontal line corresponds to the mean RLU.OD^−1^ activity before competence shift. The blue squares delimit the range of competence shift for each cell density inoculum.
(EPS)

S6 Fig Consequences of competence induction by cell contact sensing. CSP (red triangle) is exported by the dedicated ComAB transporter (green membrane proteins) allowing its capture by ComD (blue membrane protein). ComM, CbpD, CibABC, LytA are effectors of fratricide (orange scissors) [17]. CSP-mediated competence induction by cell contact requires that the potential target cell presents a compatible ComD receptor on its surface, as pictured on the left. If not, the induced fratricide proteins could attack and lyse this neighboring cell, as depicted on the right. DNA released from the killed cell could eventually be taken up by the competent attacking cell, linking genetic transformation and fratricide by cell-to-cell contact.
(EPS)

S1 Table Strains used in this study. (DOCX)

S1 Text The distinct spontaneous competence development of the CP1250 lineage. (DOCX)

## References

[1] Nealson KH, Platt T, Hastings JW. Cellular Control of the Synthesis and Activity of the Bacterial Luminescent System. J Bacteriol. 1970;104: 313–322. 5473898 10.1128/jb.104.1.313-322.1970 PMC248216

[2] Tomasz A. Model for the Mechanism Controlling the Expression of Competent State in Pneumococcus Cultures. J Bacteriol. 1966;91: 1050–1061. 4379672 10.1128/jb.91.3.1050-1061.1966 PMC315996

[3] Platt TG, Fuqua C. What’s in a name? The semantics of quorum sensing. Trends Microbiol. 2010;18: 383–387. 10.1016/j.tim.2010.05.003 20573513 PMC2932771

[4] Rutherford ST, Bassler BL. Bacterial Quorum Sensing: Its Role in Virulence and Possibilities for Its Control. Cold Spring Harb Perspect Med. 2012;2: a012427 10.1101/cshperspect.a012427 23125205 PMC3543102

[5] Waters CM, Bassler BL. QUORUM SENSING: Cell-to-Cell Communication in Bacteria. Annu Rev Cell Dev Biol. 2005;21: 319–346. 10.1146/annurev.cellbio.21.012704.131001 16212498

[6] Fuqua WC, Winans SC, Greenberg EP. Quorum sensing in bacteria: the LuxR-LuxI family of cell density-responsive transcriptional regulators. J Bacteriol. 1994;176: 269–275. 8288518 10.1128/jb.176.2.269-275.1994 PMC205046

[7] Redfield RJ. Is quorum sensing a side effect of diffusion sensing? Trends Microbiol. 2002;10: 365–370. 10.1016/S0966-842X(02)02400-9 12160634

[8] Hense BA, Kuttler C, Müller J, Rothballer M, Hartmann A, Kreft J-U. Does efficiency sensing unify diffusion and quorum sensing? Nat Rev Microbiol. 2007;5: 230–239. 10.1038/nrmicro1600 17304251

[9] Nadell CD, Bucci V, Drescher K, Levin SA, Bassler BL, Xavier JB. Cutting through the complexity of cell collectives. Proc R Soc B Biol Sci. 2013;280 10.1098/rspb.2012.2770 PMC3574390 23363630

[10] Hense BA, Schuster M. Core Principles of Bacterial Autoinducer Systems. Microbiol Mol Biol Rev. 2015;79: 153–169. 10.1128/MMBR.00024-14 25694124 PMC4402962

[11] Claverys J-P, Prudhomme M, Martin B. Induction of competence regulons as a general response to stress in gram-positive bacteria. Annu Rev Microbiol. 2006;60: 451–475. 10.1146/annurev.micro.60.080805.142139 16771651

[12] Yang J, Evans BA, Rozen DE. Signal diffusion and the mitigation of social exploitation in pneumococcal competence signalling. Proc Biol Sci. 2010;277: 2991–2999. 10.1098/rspb.2010.0659 20462905 PMC2982029

[13] Johnston C, Martin B, Fichant G, Polard P, Claverys J-P. Bacterial transformation: distribution, shared mechanisms and divergent control. Nat Rev Microbiol. 2014;12: 181–196. 10.1038/nrmicro3199 24509783

[14] Javor GT, Tomasz A. An autoradiographic study of genetic transformation. Proc Natl Acad Sci U S A. 1968;60: 1216–1222. 4386320 10.1073/pnas.60.4.1216 PMC224906

[15] Håvarstein LS, Coomaraswamy G, Morrison DA. An unmodified heptadecapeptide pheromone induces competence for genetic transformation in Streptococcus pneumoniae. Proc Natl Acad Sci. 1995;92: 11140–11144. 7479953 10.1073/pnas.92.24.11140 PMC40587

[16] Martin B, Granadel C, Campo N, Hénard V, Prudhomme M, Claverys J-P. Expression and maintenance of ComD-ComE, the two-component signal-transduction system that controls competence of Streptococcus pneumoniae. Mol Microbiol. 2010;75: 1513–1528. 10.1111/j.1365-2958.2010.07071.x 20180906

[17] Johnsborg O, Håvarstein LS. Regulation of natural genetic transformation and acquisition of transforming DNA in Streptococcus pneumoniae. FEMS Microbiol Rev. 2009;33: 627–642. 10.1111/j.1574-6976.2009.00167.x 19396959

[18] Claverys J-P, Martin B, Håvarstein LS. Competence-induced fratricide in streptococci. Mol Microbiol. 2007;64: 1423–1433. 10.1111/j.1365-2958.2007.05757.x 17555432

[19] Johnsborg O, Eldholm V, Bjørnstad ML, Håvarstein LS. A predatory mechanism dramatically increases the efficiency of lateral gene transfer in Streptococcus pneumoniae and related commensal species. Mol Microbiol. 2008;69: 245–253. 10.1111/j.1365-2958.2008.06288.x 18485065

[20] Croucher NJ, Harris SR, Fraser C, Quail MA, Burton J, van der Linden M, et al Rapid Pneumococcal Evolution in Response to Clinical Interventions. Science. 2011;331: 430–434. 10.1126/science.1198545 21273480 PMC3648787

[21] Croucher NJ, Coupland PG, Stevenson AE, Callendrello A, Bentley SD, Hanage WP. Diversification of bacterial genome content through distinct mechanisms over different timescales. Nat Commun. 2014;5 10.1038/ncomms6471 PMC4263131 25407023

[22] Hiller NL, Ahmed A, Powell E, Martin DP, Eutsey R, Earl J, et al Generation of Genic Diversity among Streptococcus pneumoniae Strains via Horizontal Gene Transfer during a Chronic Polyclonal Pediatric Infection. PLoS Pathog. 2010;6: e1001108 10.1371/journal.ppat.1001108 20862314 PMC2940740

[23] Feldman C, Anderson R. Review: Current and new generation pneumococcal vaccines. J Infect. 2014; 10.1016/j.jinf.2014.06.006 24968238

[24] Henriques-Normark B, Tuomanen EI. The pneumococcus: epidemiology, microbiology, and pathogenesis. Cold Spring Harb Perspect Med. 2013;3 10.1101/cshperspect.a010215 PMC3685878 23818515

[25] Tomasz A. Antibiotic Resistance in Streptococcus pneumoniae. Clin Infect Dis. 1997;24: S85–S88. 10.1093/clinids/24.Supplement_1.S85 8994784

[26] Prudhomme M, Claverys J-P. There will be a light: the use of luc transcriptional fusions in living pneumococcal cells The Molecular Biology of Streptococci. Norfolk: Horizon Scientific Press Hakenbeck R. and Chhatwal G.S.; 2007 pp. 519–524.

[27] Martin B, Soulet A-L, Mirouze N, Prudhomme M, Mortier-Barrière I, Granadel C, et al ComE/ComE~P interplay dictates activation or extinction status of pneumococcal X-state (competence). Mol Microbiol. 2013;87: 394–411. 10.1111/mmi.12104 23216914

[28] Dagkessamanskaia A, Moscoso M, Hénard V, Guiral S, Overweg K, Reuter M, et al Interconnection of competence, stress and CiaR regulons in Streptococcus pneumoniae: competence triggers stationary phase autolysis of ciaR mutant cells. Mol Microbiol. 2004;51: 1071–1086. 14763981 10.1111/j.1365-2958.2003.03892.x

[29] Lee MS, Morrison DA. Identification of a new regulator in Streptococcus pneumoniae linking quorum sensing to competence for genetic transformation. J Bacteriol. 1999;181: 5004–5016. 10438773 10.1128/jb.181.16.5004-5016.1999 PMC93990

[30] Peterson S, Cline RT, Tettelin H, Sharov V, Morrison DA. Gene expression analysis of the Streptococcus pneumoniae competence regulons by use of DNA microarrays. J Bacteriol. 2000;182: 6192–6202. 11029442 10.1128/jb.182.21.6192-6202.2000 PMC94756

[31] Peterson SN, Sung CK, Cline R, Desai BV, Snesrud EC, Luo P, et al Identification of competence pheromone responsive genes in Streptococcus pneumoniae by use of DNA microarrays. Mol Microbiol. 2004;51: 1051–1070. 14763980 10.1046/j.1365-2958.2003.03907.x

[32] Claverys J-P, Håvarstein LS. Cannibalism and fratricide: mechanisms and raisons d’être. Nat Rev Microbiol. 2007;5: 219–229. 10.1038/nrmicro1613 17277796

[33] Claverys J-P, Martin B, Polard P. The genetic transformation machinery: composition, localization, and mechanism. FEMS Microbiol Rev. 2009;33: 643–656. 10.1111/j.1574-6976.2009.00164.x 19228200

[34] Mirouze N, Bergé MA, Soulet A-L, Mortier-Barrière I, Quentin Y, Fichant G, et al Direct involvement of DprA, the transformation-dedicated RecA loader, in the shut-off of pneumococcal competence. Proc Natl Acad Sci U S A. 2013;110: E1035–1044. 10.1073/pnas.1219868110 23440217 PMC3600483

[35] Weng L, Piotrowski A, Morrison DA. Exit from Competence for Genetic Transformation in Streptococcus pneumoniae Is Regulated at Multiple Levels. PLoS ONE. 2013;8: e64197 10.1371/journal.pone.0064197 23717566 PMC3661451

[36] Pai A, You L. Optimal tuning of bacterial sensing potential. Mol Syst Biol. 2009;5: 286 10.1038/msb.2009.43 19584835 PMC2724973

[37] Claverys J-P, Havarstein LS. Extracellular-peptide control of competence for genetic transformation in Streptococcus pneumoniae. Front Biosci J Virtual Libr. 2002;7: d1798–1814. 10.2741/claverys 12133809

[38] Chen J-D, Morrison DA. Modulation of Competence for Genetic Transformation in Streptococcus pneumoniae. J Gen Microbiol. 1987;133: 1959–1967. 10.1099/00221287-133-7-1959 3668504

[39] Prudhomme M, Attaiech L, Sanchez G, Martin B, Claverys J-P. Antibiotic stress induces genetic transformability in the human pathogen Streptococcus pneumoniae. Science. 2006;313: 89–92. 10.1126/science.1127912 16825569

[40] Tomasz A, Mosser JL. On the nature of the pneumococcal activator substance. Proc Natl Acad Sci U S A. 1966;55: 58 4380138 10.1073/pnas.55.1.58 PMC285755

[41] Gardan R, Besset C, Gitton C, Guillot A, Fontaine L, Hols P, et al Extracellular Life Cycle of ComS, the Competence-Stimulating Peptide of Streptococcus thermophilus. J Bacteriol. 2013;195: 1845–1855. 10.1128/JB.02196-12 23396911 PMC3624564

[42] Ravin AW. Reciprocal capsular transformations of pneumococci. J Bacteriol. 1959;77: 296 13641188 10.1128/jb.77.3.296-309.1959 PMC290367

[43] Tiraby J-G, Fox MS. Marker Discrimination in Transformation and Mutation of Pneumococcus. Proc Natl Acad Sci U S A. 1973;70: 3541–3545. 4148702 10.1073/pnas.70.12.3541 PMC427276

[44] Pozzi G, Masala L, Iannelli F, Manganelli R, Håvarstein LS, Piccoli L, et al Competence for genetic transformation in encapsulated strains of Streptococcus pneumoniae: two allelic variants of the peptide pheromone. J Bacteriol. 1996;178: 6087–6090. 8830714 10.1128/jb.178.20.6087-6090.1996 PMC178474

[45] Pestova EV, Håvarstein LS, Morrison DA. Regulation of competence for genetic transformation in Streptococcus pneumoniae by an auto-induced peptide pheromone and a two-component regulatory system. Mol Microbiol. 1996;21: 853–862. 8878046 10.1046/j.1365-2958.1996.501417.x

[46] Ween O, Gaustad P, Håvarstein LS. Identification of DNA binding sites for ComE, a key regulator of natural competence in Streptococcus pneumoniae. Mol Microbiol. 1999;33: 817–827. 10447890 10.1046/j.1365-2958.1999.01528.x

[47] Dubnau D, Losick R. Bistability in bacteria. Mol Microbiol. 2006;61: 564–572. 10.1111/j.1365-2958.2006.05249.x 16879639

[48] Eldar A, Elowitz MB. Functional roles for noise in genetic circuits. Nature. 2010;467: 167–173. 10.1038/nature09326 20829787 PMC4100692

[49] Silva-Rocha R, de Lorenzo V. Noise and robustness in prokaryotic regulatory networks. Annu Rev Microbiol. 2010;64: 257–275. 10.1146/annurev.micro.091208.073229 20825349

[50] Veening J-W, Smits WK, Kuipers OP. Bistability, Epigenetics, and Bet-Hedging in Bacteria. Annu Rev Microbiol. 2008;62: 193–210. 10.1146/annurev.micro.62.081307.163002 18537474

[51] Norman TM, Lord ND, Paulsson J, Losick R. Stochastic Switching of Cell Fate in Microbes. Annu Rev Microbiol. 2015; 10.1146/annurev-micro-091213-112852 26332088

[52] Slager J, Kjos M, Attaiech L, Veening J-W. Antibiotic-induced replication stress triggers bacterial competence by increasing gene dosage near the origin. Cell. 2014;157: 395–406. 10.1016/j.cell.2014.01.068 24725406

[53] Stevens KE, Chang D, Zwack EE, Sebert ME. Competence in Streptococcus pneumoniae is regulated by the rate of ribosomal decoding errors. mBio. 2011;2 10.1128/mBio.00071-11 PMC3175624 21933920

[54] Sasková L, Nováková L, Basler M, Branny P. Eukaryotic-Type Serine/Threonine Protein Kinase StkP Is a Global Regulator of Gene Expression in Streptococcus pneumoniae. J Bacteriol. 2007;189: 4168–4179. 10.1128/JB.01616-06 17416671 PMC1913385

[55] Ibrahim YM, Kerr AR, McCluskey J, Mitchell TJ. Control of virulence by the two-component system CiaR/H is mediated via HtrA, a major virulence factor of Streptococcus pneumoniae. J Bacteriol. 2004;186: 5258–5266. 10.1128/JB.186.16.5258-5266.2004 15292127 PMC490881

[56] Laux A, Sexauer A, Sivaselvarajah D, Kaysen A, Brückner R. Control of competence by related non-coding csRNAs in Streptococcus pneumoniae R6. Front Genet. 2015;6: 246 10.3389/fgene.2015.00246 26257773 PMC4507080

[57] Cassone M, Gagne AL, Spruce LA, Seeholzer SH, Sebert ME. The HtrA protease from Streptococcus pneumoniae digests both denatured proteins and the competence-stimulating peptide. J Biol Chem. 2012;287: 38449–38459. 10.1074/jbc.M112.391482 23012372 PMC3493890

[58] Johnsborg O, Kristiansen PE, Blomqvist T, Håvarstein LS. A hydrophobic patch in the competence-stimulating Peptide, a pneumococcal competence pheromone, is essential for specificity and biological activity. J Bacteriol. 2006;188: 1744–1749. 10.1128/JB.188.5.1744-1749.2006 16484185 PMC1426553

[59] Tomasz A, Zanati E. Appearance of a protein “agglutinin” on the spheroplast membrane of pneumococci during induction of competence. J Bacteriol. 1971;105: 1213–1215. 4396142 10.1128/jb.105.3.1213-1215.1971 PMC248564

[60] Håvarstein LS, Martin B, Johnsborg O, Granadel C, Claverys J-P. New insights into the pneumococcal fratricide: relationship to clumping and identification of a novel immunity factor. Mol Microbiol. 2006;59: 1297–1307. 10.1111/j.1365-2958.2005.05021.x 16430701

[61] Marks LR, Reddinger RM, Hakansson AP. High levels of genetic recombination during nasopharyngeal carriage and biofilm formation in Streptococcus pneumoniae. mBio. 2012;3 10.1128/mBio.00200-12 PMC3448161 23015736

[62] Eldholm V, Johnsborg O, Haugen K, Ohnstad HS, Håvarstein LS. Fratricide in Streptococcus pneumoniae: contributions and role of the cell wall hydrolases CbpD, LytA and LytC. Microbiol Read Engl. 2009;155: 2223–2234. 10.1099/mic.0.026328-0 19389766

[63] Guiral S, Mitchell TJ, Martin B, Claverys J-P. Competence-programmed predation of noncompetent cells in the human pathogen Streptococcus pneumoniae: genetic requirements. Proc Natl Acad Sci U S A. 2005;102: 8710–8715. 10.1073/pnas.0500879102 15928084 PMC1150823

[64] Kausmally L, Johnsborg O, Lunde M, Knutsen E, Håvarstein LS. Choline-Binding Protein D (CbpD) in Streptococcus pneumoniae Is Essential for Competence-Induced Cell Lysis. J Bacteriol. 2005;187: 4338–4345. 10.1128/JB.187.13.4338-4345.2005 15968042 PMC1151764

[65] Moscoso M, Claverys J-P. Release of DNA into the medium by competent Streptococcus pneumoniae: kinetics, mechanism and stability of the liberated DNA. Mol Microbiol. 2004;54: 783–794. 10.1111/j.1365-2958.2004.04305.x 15491367

[66] Steinmoen H, Knutsen E, Håvarstein LS. Induction of natural competence in Streptococcus pneumoniae triggers lysis and DNA release from a subfraction of the cell population. Proc Natl Acad Sci. 2002;99: 7681–7686. 10.1073/pnas.112464599 12032343 PMC124321

[67] Håvarstein LS, Hakenbeck R, Gaustad P. Natural competence in the genus Streptococcus: evidence that streptococci can change pherotype by interspecies recombinational exchanges. J Bacteriol. 1997;179: 6589–6594. 9352904 10.1128/jb.179.21.6589-6594.1997 PMC179583

[68] Johnsborg O, Blomqvist T, Mogens K, Håvarstein LS. Biogically active peptides in Streptococci Molecular Biology of Strpetococci. Norfolk: Horizon Scientific Press Hakenbeck R. and Chhatwal G.S.; 2007 pp. 25–59.

[69] Tomasz A, Hotchkiss RD. REGULATION OF THE TRANSFORMABILITY OF PNEUMOCOCCAL CULTURES BY MACROMOLECULAR CELL PRODUCTS. Proc Natl Acad Sci U S A. 1964;51: 480 14171462 10.1073/pnas.51.3.480 PMC300099

[70] Martin B, Prudhomme M, Alloing G, Granadel C, Claverys JP. Cross-regulation of competence pheromone production and export in the early control of transformation in Streptococcus pneumoniae. Mol Microbiol. 2000;38: 867–878. 11115120 10.1046/j.1365-2958.2000.02187.x

[71] SICARD AM. A NEW SYNTHETIC MEDIUM FOR DIPLOCOCCUS PNEUMONIAE, AND ITS USE FOR THE STUDY OF RECIPROCAL TRANSFORMATIONS AT THE AMIA LOCUS. Genetics. 1964;50: 31–44. 14191356 10.1093/genetics/50.1.31 PMC1210647

[72] Ottolenghi E, Hotchkiss RD. Release of genetic transforming agent from pneumococcal cultures during growth and disintegration. J Exp Med. 1962;116: 491–519. 13940741 10.1084/jem.116.4.491 PMC2137623

