# Supplementary material for: Pneumococcal Competence Coordination Relies on a Cell-Contact Sensing Mechanism
Source: PLoS Genet. 2016 Jun 29;12(6):e1006113. doi: 10.1371/journal.pgen.1006113 (PMC4927155; doi:10.1371/journal.pgen.1006113)
Supplement: S1 Table — (DOCX) [file pgen.1006113.s007.docx]

Tables

**Table S1: Strains used in this study**

| Strain | Genotype^a^/description | source |
| --- | --- | --- |
| R800 | Wild type representative of this lineage | [75] |
| R825 | R800 but *comC*::*luc* (pR414), *comC+*; Ery^R^ | [76] |
| R895 | R800 but *ssbB*::*luc* (pR424), *ssbB+*; Cm^R^ | [77] |
| TCP1251 | CP1250 but *ssbB*::*luc* (pR424), *ssbB+*; Cm^R^ | This study |
| TG55 | PR212(G54 derivative ), Δ*cps*- *aliA* ::*Kan* , but *ssbB*::*luc* (pR424), ssbB+; Cm^R^ ; Kan^R^ | This study |
| TD82 | *D39 derivative* Δ*cps::kan* but *ssbB*::*luc* (pR424), *ssbB+*; Cm^R^; Kan^R^ | [78] |
| R1313 | R895 but *comA*::*kan*42; *ssbB+*; Cm^R^ ; Kan^R^ | [39] |
| R1625 | R800 but *comA*::*kan42*; Kan^R^ | This study |
| R1745 | R800 but *comD*::*kan105*; Kan^R^ | [27] |
| R2977 | R800 but Δ*comCD comE^D58E^ rpsL41* ; Sm^R^ | [27] |

^R^, resistance; Cm, chloramphenicol; Kan, kanamycin; Sm, streptomycin. Ery, erythromycin.

#### References

75. Lefevre JC, Claverys JP, Sicard AM. Donor deoxyribonucleic acid length and marker effect in pneumococcal transformation. J Bacteriol. 1979;138: 80–86.

76. Bergé M, Moscoso M, Prudhomme M, Martin B, Claverys J-P. Uptake of transforming DNA in Gram-positive bacteria: a view from Streptococcus pneumoniae. Mol Microbiol. 2002;45: 411–421.

77. Chastanet A, Prudhomme M, Claverys JP, Msadek T. Regulation of Streptococcus pneumoniae clp genes and their role in competence development and stress survival. J Bacteriol. 2001;183: 7295–7307. doi:10.1128/JB.183.24.7295-7307.2001

78. Caymaris S, Bootsma HJ, Martin B, Hermans PWM, Prudhomme M, Claverys J-P. The global nutritional regulator CodY is an essential protein in the human pathogen Streptococcus pneumoniae. Mol Microbiol. 2010;78: 344–360.
