# Supplementary material for: Pneumococcal Competence Coordination Relies on a Cell-Contact Sensing Mechanism
Source: PLoS Genet. 2016 Jun 29;12(6):e1006113. doi: 10.1371/journal.pgen.1006113 (PMC4927155; doi:10.1371/journal.pgen.1006113)
Supplement: S1 Text — (DOCX) [file pgen.1006113.s008.docx]

### Supporting Information.

#### The distinct spontaneous competence development of the CP1250 lineage.

CP1250 is derived from the D39 strain (Figure S1). Its genome exhibits up to 6 600 single nucleotide polymorphisms in the open reading frames alone in comparison to the other parental lineages [71]. This can be attribute to both the high mutation rate due to mismatch repair deficiency and phenotypic selection pressure apply by laboratory to maintain high efficiency of natural transformation. Others reasons that could explain the distinct behavior of the CP1250 lineage are further presented in the supporting information.

The CP1250 lineage has inherited the mutation inactivating mismatch repair from the ancestor strain Rx in 1959 (Figure S1). Recent whole genome sequencing has revealed 6600 single nucleotide polymorphisms in ORFs in comparison to R6, 3768 of them not silent [71]. Some of these mutations could explain the altered competence development observed. In support to this proposal, we have previously found that expression of the late competence gene *dpr*A, whose gene product is involved in competence shut-off [34,35] via its physical interaction with the ComE-P activator (Figure 1B), is delayed in CP1250 [27]. The extended period of early gene expression in CP1250 may result in an increase in production and export of CSP. This export may exceed the capacity of cells to retain CSP and explain the presence of free CSP in the growth medium during competence development.

The X_A_ value of the pre-competence period of the CP1250 strain is particularly sensitive to the initial cell density of the culture. By contrast, the R800 strain exhibits a constant X_A_ value over a large range of inoculum concentrations (Figures 2 and S5). An R800 mutant, R1555 (also named RT, for ‘Reduced Transformation’ phenotype) contains a point mutation upstream of the *comCDE* operon. Interestingly this mutant exhibits a reduced level of spontaneous competence development, a “competence down” phenotype that we interpret as a failure to produce a sufficient amount of induced cells via an autocrine mode [72]. The variability of the X_A_ value is linked to the size of inoculum and is directly is correlated to transcription readtrough of the *comCDE* operon. This modifies the balance of the core sensor ComABCDE. Introduction of a mutation leading to overexpression of *comAB* in R1555 suppresses the RT phenotype [72], probably by restoring the balance of ComABCDE core sensor. These observations imply that alterations in the relative levels of *com*CDE and *com*AB expression influence the function of the ComABCDE core sensor and modify the X_A_ value of the pre-competence period.

#### References

71. Tovpeko Y, Morrison DA. Competence for Genetic Transformation in Streptococcus pneumoniae: Mutations in σA Bypass the comW Requirement. J Bacteriol. 2014; JB.01933–14. doi:10.1128/JB.01933-14

72. Guiral S, Hénard V, Granadel C, Martin B, Claverys J-P. Inhibition of competence development in Streptococcus pneumoniae by increased basal-level expression of the ComDE two-component regulatory system. Microbiol Read Engl. 2006;152: 323–331. doi:10.1099/mic.0.28425-0
